# Anti-CD5 CAR-T cells with a tEGFR safety switch exhibit potent toxicity control

**DOI:** 10.1038/s41408-024-01082-y

**Published:** 2024-06-18

**Authors:** Haolong Lin, Jiali Cheng, Li Zhu, Yuhao Zeng, Zhenyu Dai, Yicheng Zhang, Xiaojian Zhu, Wei Mu

**Affiliations:** 1 Department of Hematology, Tongji Hospital, Tongji Medical College, Huazhong University of Science and Technology, Wuhan, Hubei 430030 China; 2 Immunotherapy Research Center for Hematologic Diseases of Hubei Province, Wuhan, Hubei 430030 China; 3 Department of Internal Medicine, Cleveland Clinic, Akron General, Akron, OH USA; 4 Division of Blood and Marrow Transplantation and Cellular Therapy, Department of Medicine, Stanford University, Stanford, CA USA

**Keywords:** Cancer immunotherapy, Cancer immunotherapy

Dear Editor,

The dire prognosis of relapsed or refractory (r/r) aggressive T-cell malignancies underscores an urgent need for improved therapeutic strategies [1]. The efficacy of chimeric antigen receptor (CAR) modified T-cell therapy has profoundly impacted the treatment landscape for hematologic malignancies. Its success in B cell and plasma cell tumors is gradually extending to T cell malignancies. Specifically, CAR-T cells targeting CD5 and CD7, has shown promise in treating T-cell malignancies [2, 3]. However, patients often suffer from adverse events, including cytokine release syndrome (CRS), immune effector cell-associated neurotoxicity syndrome (ICANS), virus reactivation, and T-cell aplasia post-CAR-T cell infusion [4–6]. These complications present substantial challenges to the wider application of CAR-T cell therapy in T-cell malignancies, necessitating the development of safety switch systems to attenuate potentially life-threatening side effects and to restore immune function after tumor elimination.

Investigations into small molecule-based safety switches to modulate CAR-T cell activity and control toxicities post-treatment have been conducted [7]. Clinically, inducible caspase 9 (iCasp9) has proven effective in mitigating graft versus host disease (GVHD) post haploidentical stem cell transplantation [8] and in managing anti-CD19 CAR-T cell-associated neurotoxicity [9]. Ganciclovir (GCV) activated switch-off system, utilizing the herpes simplex virus-1 thymidine kinase (HSV-TK) protein, has been tested in allogeneic hematopoietic stem cell transplantation (allo-HSCT). Nevertheless, the relatively delayed clearance of donor T cells and concerns about immunogenicity limit its application potential [10]. More sophisticated switching strategies, such as the reversible on/off switch controlled by lenalidomide, have also been developed. While their safety and efficacy have yet to be validated in humans [11]. Antibody-dependent safety switch, notably the truncated epidermal growth factor receptor (tEGFR), has been preclinically tested for CAR-T cell elimination in murine models and demonstrated comparable efficacy [12, 13]. However, the efficacy of cetuximab-dependent CAR-T cell clearance in clinical settings remains uncertain. This letter reports the first clinical application of cetuximab to constrain CAR-T cell proliferation and mitigate associated side effects in patients with T cell lymphoma post anti-CD5 CAR-T cell treatment.

The three patients reported here were part of the Investigator-Initiated Trial (IIT) (NCT04767308) that evaluated the safety and efficacy of genetically edited autologous anti-CD5 CAR-T cells in the treatment of r/r CD5 positively expressing hematologic malignancies. Patient 1 is a 47-year-old female who was diagnosed with angioimmunoblastic T cell lymphoma (AITL) and had progressive disease (PD) status at enrollment. Patient 2 is a 49-year-old male with AITL and had stable disease (SD) status at enrollment. Patient 3 is a 31-year-old female with subcutaneous panniculitis-like T cell lymphoma (SPTCL) and had a PD status at enrollment (Table S1). The CAR construct comprised biepitopic, fully human-derived CD5-targeting nanobodies, 4-1BB costimulatory element, CD3ζ activation domains, and a tEGFR switch connected by a peptide of Thosea asigna virus [2]. The manufacturing process of anti-CD5 CAR-T cells, including leukapheresis collection, T cell isolation and activation, Cas9 ribonucleoprotein (RNP) mediated *CD5* gene deletion, CAR gene-carrying lentivirus transduction, CAR-T cell expansion, formulation and cryopreservation is shown in Fig. S1. This study was approved by the institutional review board of Tongji Hospital, Tongji Medical College, Huazhong University of Science and Technology, and the assessments of adverse effects were conducted strictly according to the National Cancer Institute (NCI) and American Society for Transplantation and Cellular Therapy criteria (ASTCT). In this study, the kinetics of CAR-T cells in peripheral blood were monitored by drop digital polymerase chain reaction (ddPCR) and flow cytometry. Natural killer (NK) cells and neutrophils were detected by flow cytometry, and inflammatory cytokines and acute phase proteins in plasma were detected by chemiluminescence and turbidimetric inhibition immunoassay respectively.

These three patients, each with a history of multiple lines of prior therapy, received lymphodepleting chemotherapy followed by anti-CD5 CAR-T cell infusion at a dose of 1.0 × 10^6^/kg (Table S1). Details of infused CAR-T cell products, including the percentage of T cells and CAR-T cells, viability and cytotoxicity of CAR-T cells, and microbial test for each product were listed in Table S2. CAR-T cells in three patients all had a rapid in vivo expansion post infusion. Subsequently, all three patients developed grade 1 CRS. Around two weeks post infusion, when the CAR-T cells expansion was substantial, with the copy number reaching 76,645/43,123/112,000 per microgram of DNA for patients 1–3, respectively, the patients developed severe rash, skin tingling and other skin-related symptoms (Fig. 1). Given the persistence of grade 3–4 rash and tingling despite standard care and corticosteroids, cetuximab was introduced at a dose of 100 mg or 200 mg to these patients. Remarkably, the CAR transgene numbers exhibited a significant decline, reducing from 76,645 to 1468 copies/μg, 43,123 to 927 copies/μg and 112,000 to 15,641 copies/μg in patients 1–3, respectively within seven days after cetuximab administration (Fig. 1A). Percentage of CAR-T cells in T cells population also decreased from 61.35% to 15.97%, 50.95% to 14.09%, and 60.85% to 21.29% in peripheral blood (Fig. 1B). Absolute number of CAR-T cells in peripheral blood declined from 15.58 × 10^7^ to 0.22 × 10^7^, 3.75 × 10^7^ to 0.11 × 10^7^ and 12.40 × 10^7^ to 1.05 × 10^7^ per liter (Table S3). Simultaneously, skin-related symptoms were relieved (Fig. 1C). However, even after this intervention, low levels of CAR transgene remained detectable about 100 days post CAR-T cell infusion (unpublished data). While the clinical outcomes were not favorable. Patient 1 achieved complete remission (CR) but died of sepsis and multi-organ dysfunction on day 124 post infusion. Patient 2 underwent allo-HSCT at day 102 after achieving partial remission (PR), and patient 3 relapsed on day 182 after achieving PR (Table S1).

**Fig. 1:**
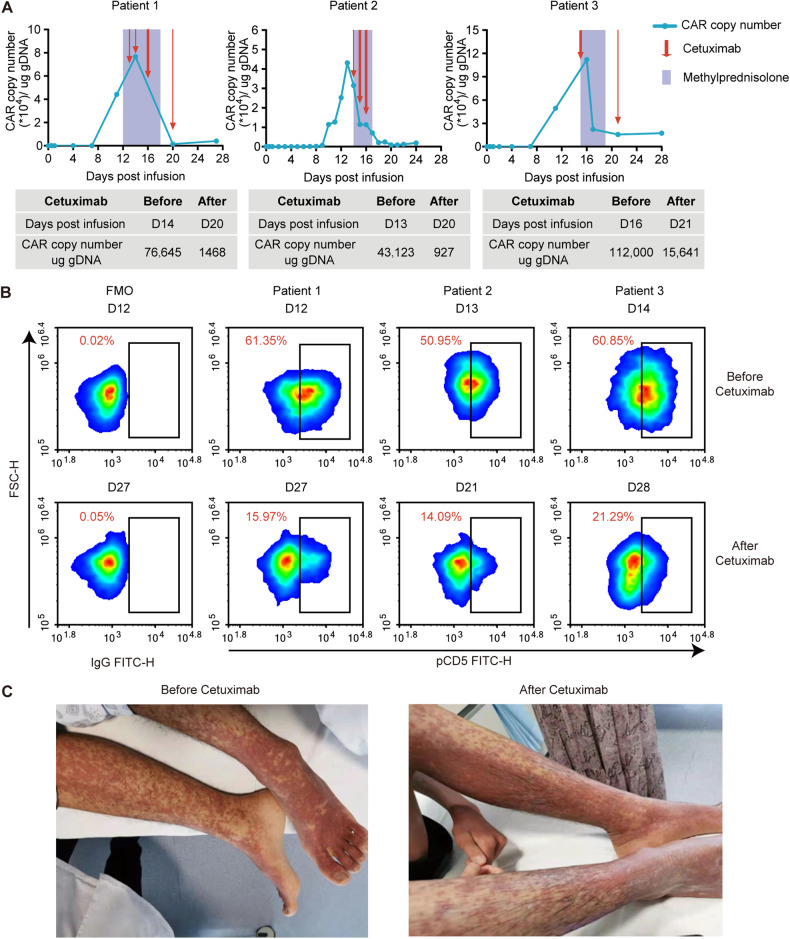
Levels of tEGFR-transduced anti-CD5 CAR-T cells in peripheral blood and representative rash during cetuximab administration. **A** Copy number of CAR transgene in peripheral blood monitored through ddPCR following anti-CD5 CAR-T cell infusion within 28 days. The CAR transgene numbers exhibited a significant decline, reducing from 76,645 to 1468 copies/μg, 43,123 to 927 copies/μg, and 112,000 to 15,641 copies/μg in patients 1–3, respectively, within seven days after cetuximab administration. Each red arrow indicates a single administration of cetuximab, with thick arrows representing a dose of 200 mg and thin arrows representing a dose of 100 mg. The purple shaded intervals indicate the duration of continuous methylprednisolone administration during the specified periods: days 12 to 18 post-infusion for patient 1, days 14 to 17 post-infusion for patient 2, and days 15 to 19 post-infusion for patient 3. **B** Percentage of anti-CD5 CAR-T cells in the three patients evaluated by flow cytometry analysis before (top row) and after (bottom row) cetuximab administration. The percentage of CAR-T cells in T cells of peripheral blood also decreased from 61.35% to 15.97%, 50.95% to 14.09%, and 60.85% to 21.29%. The numbers shown in the panel were gated from CD3^+^ T cells. Fluorescein isothiocyanate-labeled human CD5 protein was used to detect CAR-positive cells. **C** A representative image of the rash in patient 2 before and after cetuximab administration, showing skin-related symptoms were relieved.

Despite the vigorous proliferation of CAR-T cells and the occurrence of skin-related symptoms, serum cytokines, including IL-6, IL-1β, IL-10, IL-8, and TNF-α remained relatively stable, suggesting that the skin symptoms were not related to cytokines dysregulation. Acute phase proteins, including ferritin and hypersensitive C-reactive protein (hsCRP), were all above the normal range for 28 days after infusion, except for patient 1, in whom ferritin remained in the normal range (30–400 μg/L) (Fig. 2A).

**Fig. 2:**
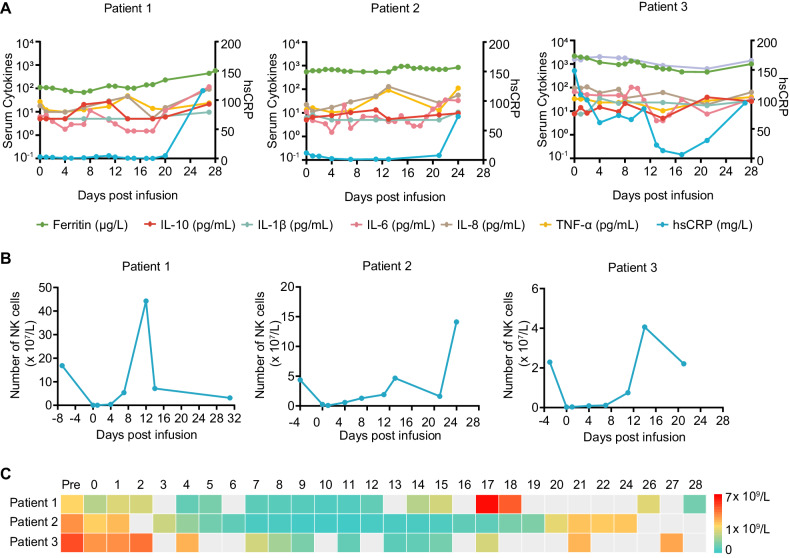
Concentration of serum cytokines, number of NK cells, and neutrophils in peripheral blood at the indicated time points after CAR-T cell infusion. **A** Concentration of cytokines, including IL-10 (ranging between 5.0–26.7 pg/mL, 5.0–13.3 pg/mL, and 5.0–38.8 pg/mL in patients 1–3, respectively), IL-1β (ranging between 5.0–9.6 pg/mL, 5.0–10.7 pg/mL, and 7.5–35.4 pg/mL in patients 1–3, respectively), IL-6 (ranging between 1.5–116.7 pg/mL, 1.5–34.9 pg/mL and 3.95–103.3 pg/mL in patients 1–3, respectively), IL-8 (ranging between 5.3–92.3 pg/mL, 11.5–129.0 pg/mL, and 19.1–107.0 pg/mL in patients 1–3, respectively), and TNF-α (ranging between 9.8–48.1 pg/mL, 10.4–111.0 pg/mL, and 10.4–39.5 pg/mL in patients 1–3, respectively), as well as two acute phase proteins, ferritin (ranging between 67.7–573.5 μg/L, 539.8–935.9 μg/L, and 459.6–2181.2 μg/L in patients 1–3, respectively) and hypersensitive C-reactive protein (ranging between 0.1–116.0 mg/L, 1.0–73.8 mg/L, and 6.5–148.6 mg/L in patients 1–3, respectively), at various time points after CAR-T cell infusion. Note: Detection limits for IL-10, IL-1β, and IL-6 are 5.0 pg/mL, 5.0 pg/mL, and 1.5 pg/mL, respectively, when concentrations fall below these limits, the default value is set to the detection limit. All other values in the figure are within the detectable range; **B**, **C** Absolute numbers of NK cells (**B**) and neutrophils (**C**) in the peripheral blood of the three patients within 28 days post-infusion. During cetuximab administration in three patients, the number of NK cells per liter was 7.12 × 10^7^ at day 14, 4.67 × 10^7^ at day 13, and 2.21 × 10^7^ at day 21. The number of neutrophils ranged between 0.62 × 10^9^–4.47 × 10^9^, 0.05 × 10^9^–0.16 × 10^9^, and 0.27 × 10^9^–2.04 × 10^9^.

In principle, the effectiveness of cetuximab is contingent upon the patient’s immune effector cells, notably NK cells and neutrophils. However, markedly low levels of these cells were observed (Fig. 2B, C). During cetuximab administration in three patients (Day 13–20 for patient 1, Day 14–16 for patient 2, Day 15–21 for patient 3), the number of NK cells per liter was 7.12 × 10^7^ at day 14, 4.67 × 10^7^ at day 13 and 2.21 × 10^7^ at day 21 (Fig. 2B) and the number of neutrophils ranged between 0.62 × 10^9^–4.47 × 10^9^, 0.05 × 10^9^–0.16 × 10^9^ and 0.27 × 10^9^–2.04 × 10^9^, respectively (Fig. 2C). These indicated that cetuximab can function even when the number of NK cells and neutrophils are as low as 2.21 × 10^7^ and 0.05 × 10^9^ per liter. Notably, administration of cetuximab did not significantly affect these cell counts, nor did it cause additional adverse events.

In conclusion, this study demonstrates that anti-CD5 CAR-T cells, engineered with a tEGFR safety switch, can be effectively diminished using cetuximab. The rapid decline of CAR-T cells in peripheral blood and alleviation of side effects within seven days of cetuximab administration underscores its immediate impact. Notably, the decline rate of CAR-T cells after reaching their crest was much slower in patients who did not receive cetuximab in this trial. Furthermore, it’s noteworthy that the administration of corticosteroids has been demonstrated to be ineffective in limiting the kinetics of CAR-T cells [14, 15]. Importantly, although more than 90% of the CAR-T cells were eliminated after cetuximab, the remaining CAR-T cells can provide anti-tumor surveillance and induce promising therapeutic efficacy.

In our study, we highlighted the occurrence of skin-associated adverse events following genetically edited autologous anti-CD5 CAR-T cell therapy. These cutaneous manifestations emerged concurrently with the robust proliferation of CAR-T cells and subsided with their decline. Notably, these skin symptoms arose without significant fluctuations in serum cytokine levels, including IL-6 and ferritin, which typically increased during the period of cytokine release syndrome. This observation suggests that the pathogenesis of skin discomfort or rash after anti-CD5 CAR-T cell infusion might operate independently of cytokine release, potentially attributable to the on-target, off-tumor effects of anti-CD5 CAR-T cells or T cell dysregulation. This warrants further detailed investigation to deepen our understanding. Our findings highlight the necessity of integrating a switch-off system in the design of novel CAR-T cell products. Moreover, considering the dose-dependent and immune cell-dependent proapoptotic effects of cetuximab, its therapeutic efficacy could be optimized by modulating the dosage to balance the antitumor response against the adverse effects.

Overall, the phenomena of on-target, off-tumor toxicity and immune dysfunction subsequent to CAR-T cell therapy pose significant challenges in the realm of advanced CAR-T cell therapeutics. The integration of a switch-off feature, as exemplified in our clinical data utilizing tEGFR, offers a promising approach to augment the safety profile of cellular immunotherapies.

### Supplementary information

Supplemental materials

## Data Availability

The data generated and/or analyzed during the course of this study are available from the corresponding author upon reasonable request.
