# Correction: Anti-CD5 CAR-T cells with a tEGFR safety switch exhibit potent toxicity control

**DOI:** 10.1038/s41408-024-01094-8

**Published:** 2024-07-24

**Authors:** Haolong Lin, Jiali Cheng, Li Zhu, Yuhao Zeng, Zhenyu Dai, Yicheng Zhang, Xiaojian Zhu, Wei Mu

**Affiliations:** 1 Department of Hematology, Tongji Hospital, Tongji Medical College, Huazhong University of Science and Technology, Wuhan, Hubei 430030 China; 2 Immunotherapy Research Center for Hematologic Diseases of Hubei Province, Wuhan, Hubei 430030 China; 3 Department of Internal Medicine, Cleveland Clinic, Akron General, Akron, OH USA; 4 Division of Blood and Marrow Transplantation and Cellular Therapy, Department of Medicine, Stanford University, Stanford, CA USA

**Keywords:** Cancer immunotherapy, Cancer immunotherapy

Correction to: *Blood Cancer Journal* 10.1038/s41408-024-01082-y, published online 18 June 2024

In the original article the following changes has been implemented

- In the 6th paragraph, lines 6 and 12, “per microliter” should be changed to “per liter.”
- In Fig. 2B, the legend on line 12 should also change from “per microliter” to “per liter.”
- The *y*-axis labels for NK cells in Fig. 2B should be expressed in units of 10^7^/L.

