# Supplementary material for: Anti-CD5 CAR-T cells with a tEGFR safety switch exhibit potent toxicity control
Source: Blood Cancer J. 2024 Jun 18;14(1):98. doi: 10.1038/s41408-024-01082-y (PMC11189405; doi:10.1038/s41408-024-01082-y)
Supplement: Supplementary file 1 — Supplemental materials [file 41408_2024_1082_MOESM1_ESM.docx]

**Supplemental materials**

**Study Design**

The open-label, single-center Investigator-Initiated Trial (IIT) employed a "3+3" dose-escalation design and was conducted at Department of Hematology, Tongji Hospital, starting on February 1, 2021. Three dosage levels were set at 1x10^6^/kg, 2 x10^6^/kg, and 3 x10^6^/kg, with each cohort expected to enroll one to six subjects, totaling enrollment of 6 to 18 patients. The primary objectives of this trial included assessing the safety of autologous anti-CD5 CAR-T products (CT125A) in subjects with relapsed/refractory (r/r) CD5^+^ hematologic tumors and determining the maximum tolerated dose (MTD). The secondary goal was to evaluate the effectiveness of CT125A in patients with r/r CD5^+^ hematologic tumors. The clinical trial was conducted in strict accordance with the Declaration of Helsinki, with approval and consent from the Ethics Committee of Tongji Medical College (NCT04767308).

Patients who volunteered to participate in this trial were fully informed the trial’s details and provided signed informed consent forms. Then, they underwent the screening process.

The eligibility criteria:

1) Participants with CD5-positive r/r B-cell lymphomas including chronic lymphocytic leukemia (CLL)/small lymphocytic lymphoma (SLL) resistant to BTK inhibitor, mantle cell lymphoma unresponsive to anthracyclines, bendamustine, CD20 monoclonal antibodies, or BTK inhibitor, and DLBCL unsuitable for autologous hematopoietic transplantation (HSCT) or relapsed after HSCT, were included. Patients needed to have at least one measurable lesion exceeding 1.5 cm in length, or bone marrow (BM) involvement confirmed by flow cytometry (FCM).

2) Participants with CD5-positive r/r peripheral T-cell lymphomas, each having at least one measurable lesion exceeding 1.5 cm in length, without BM invasion confirmed both by FCM and TCR/IGH receptor gene rearrangement assay.

3) Additional criteria included: age between 18 and 70 years; survival expectancy ≥ 12 weeks; ECOG performance status < 2; left ventricular ejection fraction ≥ 50%; blood oxygen saturation > 91%; alanine aminotransferase and aspartate aminotransferase levels ≤ 2.5 times of upper limit of normal (ULN); total serum bilirubin levels ≤ 37.2 μmol/L (≤3.0 ULN for Gilbert's syndrome); estimated glomerular filtration rate (GFR) by CKD-EPI formula ≥ 30 ml/min/1.73m^2^; agreement to use effective contraception for a year post CAR-T cell infusion; and a negative serum or urine pregnancy test for women of childbearing age.

Patients meeting any of the following exclusion criteria should be excluded:

1. A history of allergic reactions to any components of the cell product.
2. GVHD ≥ grade II as per Glucksberg standards or ≥ severity B according to IBMTR, or requiring systemic treatment within four weeks prior to enrollment.
3. Receipt of live vaccines within four weeks preceding enrollment.
4. Presence of life-threatening central nervous system diseases, such as cerebral aneurysms, epilepsy, stroke, dementia, or psychosis.
5. Severe active infections requiring intravenous antibiotic treatment.
6. Positive hepatitis B surface antigen (HBsAg) or hepatitis B core antibody (HBcAb) with peripheral blood hepatitis B virus (HBV) DNA levels exceeding 100 IU/mL.
7. Positive hepatitis C virus (HCV) antibody and positive peripheral blood HCV RNA.
8. Other immunodeficiencies, including HIV infection, and cytomegalovirus (CMV) DNA levels exceeding 400 copies/mL.
9. History of other primary cancers, except for cured non-melanoma skin cancer such as basal cell carcinoma, cured in situ cancers such as cervical, bladder, or breast cancer, or other primary cancers with no recurrence for more than 5 years.
10. A history of solid organ transplantation.
11. Autoimmune diseases, requiring immunosuppressive therapy.
12. Participation in other interventional clinical trials within three months before signing the informed consent form.
13. Breastfeeding status.
14. Mental illness, or altered mental status.
15. Unresolved severe toxicity (>grade 2 according to NCI-CTCAE v5.0) from prior treatments, except for hair loss.
16. Administration of therapeutic doses of steroids within 72 hours prior CAR-T infusion, or systemic antitumor treatments within two weeks or five half-lives prior to leukapheresis.

After screening, patients prepared for the leukapheresis, and the manufacture of CAR-T cells started. During this period, bridging therapy was permitted for patients with rapidly progressing disease. Before the infusion of the prescribed dose of CT125A, patients received a 3-day pre-conditioning regimen of FC (fludarabine 30mg/m^2^/day, i.v.; cyclophosphamide 500mg/m^2^/day, i.v.) followed by one-day of rest. Patients who received treatment were subjected to a 2-year primary follow-up for safety and efficacy observation, a 15-year long-term follow-up for monitoring secondary tumor occurrence and long-term treatment effectiveness, or until the patient withdrew their consents.

**Supplementary Figure 1**


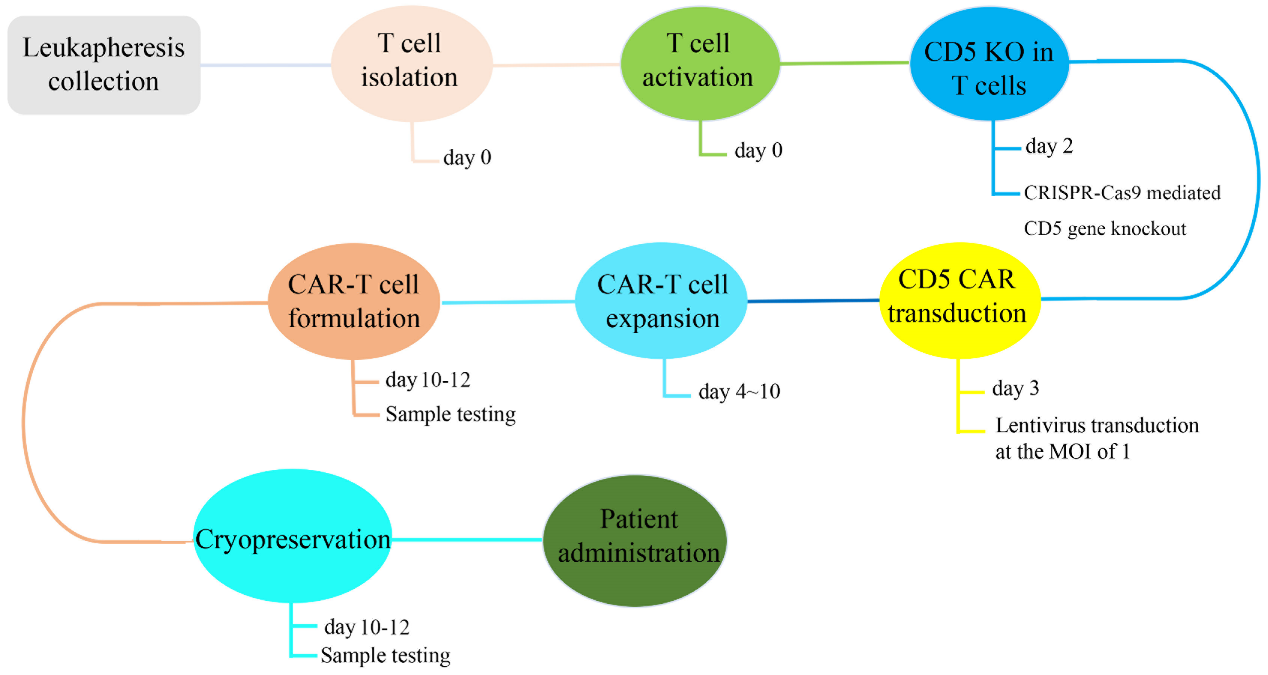


Supplementary Figure 1: Manufacturing process of CD5 CAR-T cells. Patients underwent leukapheresis for peripheral blood mononuclear cell (PBMC) collection. CD3 positive T cells were isolated by using pan T cell isolation kit (Miltenyi) and activated by MACS GMP T cell TransActTM at day 0. On day 2, 4µg Cas9 protein (Kactus) and 2µg CD5 sgRNA (GenScript) per 1x10^6^ cells were electroporated using Celetrix electroporation. About 18-24h post electroporation, Lentivirus carrying CD5 CAR structure was transduced at the MOI of one. After six days of expansion *in vitro* in CTS medium supplemented with IL-2 and L-glutamine, CAR-T cells were formulated on day 10 and underwent sample testing, which included measurement of the percentage of CD3^+^CAR^+^ and CD3^+^CD5^-^ cells, cell viability, appearance, osmotic pressure, copy number of CAR transgenes, sterility, endotoxin and mycoplasma levels. Additionally, IFN-γrelease were measured to evaluated the function of CD5 CAR-T cells when encountering CD5^+^ Jurkat cell line. The CAR-T cell products were then cryogenically frozen on day 12 and prepared for patient administration.

**Supplementary Table 1** Clinical characteristics of enrolled patients and therapeutic outcomes

|  | **Patient 1** | **Patient 2** | **Patient 3** |
| --- | --- | --- | --- |
| Age (yr) | 47 | 49 | 31 |
| Sex | F | M | F |
| Weight (kg) | 51 | 68 | 52 |
| Diagnosis | AITL | AITL | SPTCL |
| Prior lines of therapy | 2 | 3 | 6 |
| Disease status at enrollment | PD | SD | PD |
| Dose | 1.0×10^6^/kg | 1.0×10^6^/kg | 1.0×10^6^/kg |
| Clinical outcome | CR and died at day 124**^#^** | PR and had allo-HSCT at day 102* | PR and relapsed at day 182 |

Note. yr, years; F, female; M, male; kg, kilogram; AITL: angioimmunoblastic T cell lymphoma; SPTCL: subcutaneous panniculitis-like T-cell lymphoma; PD: progressed disease; SD: stable disease; CR: complete remission; PR: partial remission; Allo-HSCT: allogeneic hematopoietic stem cell transplantation;
*The patient had persistent severe myelosuppression and received allo-HSCT after remission.
#: The patient died of sepsis and multi-organ dysfunction.

**Supplementary Table 2** Characteristics of CAR-T cell products for each patient enrolled

| **ID** | **CD3+%** | **CAR+%** | **Viability** | **IFN-γ or Cytotoxicity** | **Microbial testing** |
| --- | --- | --- | --- | --- | --- |
| Patient 1 | 98.52% | 27.60% | 78.43% | 12.89 fg/CD3^+^CAR^+^ | Negative |
| Patient 2 | 99.58% | 21.55% | 73.66% | 17.95 fg/CD3^+^CAR^+^ | Negative |
| Patient 3 | 96.40% | 49.60% | 95.05% | 15.86 fg/CD3^+^CAR^+^ | Negative |
| **Criteria** | > 90% | > 10% | > 70% | N/A | Negative |

**Supplementary Table 3** Absolute number of CAR-T cells in peripheral blood before and after cetuximab administration

| **ID** | **Days post CD5 CAR-T cell infusion** | **Lymphocyte counts**  **(10^9^/L)** | **Percent of CAR-T cell in lymphocyte (%)** | **Absolute CAR-T cell numbers (10^7^/L)** |
| --- | --- | --- | --- | --- |
| Patient 1 | Day 12 | 0.78 | 19.97 | 15.58 |
|  | Day 27 | 0.03 | 7.43 | 0.22 |
| Patient 2 | Day 13 | 0.14 | 26.81 | 3.75 |
|  | Day 21 | 0.03 | 3.65 | 0.11 |
| Patient 3 | Day 14 | 1.22 | 10.16 | 12.40 |
|  | Day 28 | 1.33 | 0.79 | 1.05 |
